# A Mouse Systems Genetics Approach Reveals Common and Uncommon Genetic Modifiers of Hepatic Lysosomal Enzyme Activities and Glycosphingolipids

**DOI:** 10.3390/ijms24054915

**Published:** 2023-03-03

**Authors:** Anyelo Durán, David A. Priestman, Macarena Las Heras, Boris Rebolledo-Jaramillo, Valeria Olguín, Juan F. Calderón, Silvana Zanlungo, Jaime Gutiérrez, Frances M. Platt, Andrés D. Klein

**Affiliations:** 1 Centro de Genética y Genómica, Facultad de Medicina, Clínica Alemana Universidad del Desarrollo, Santiago 7610658, Chile; 2 Department of Pharmacology, University of Oxford, Oxford OX1 3QT, UK; 3 Research Center for the Development of Novel Therapeutic Alternatives for Alcohol Use Disorders, Santiago 7610658, Chile; 4 Department of Gastroenterology, Faculty of Medicine, Pontificia Universidad Católica de Chile, Santiago 8330033, Chile; 5 Cellular Signaling and Differentiation Laboratory, School of Medical Technology, Health Sciences Faculty, Universidad San Sebastian, Santiago 7510602, Chile

**Keywords:** metabolism, lysosomal enzymes, glycosphingolipids, systems genetics, modifier genes

## Abstract

Identification of genetic modulators of lysosomal enzyme activities and glycosphingolipids (GSLs) may facilitate the development of therapeutics for diseases in which they participate, including Lysosomal Storage Disorders (LSDs). To this end, we used a systems genetics approach: we measured 11 hepatic lysosomal enzymes and many of their natural substrates (GSLs), followed by modifier gene mapping by GWAS and transcriptomics associations in a panel of inbred strains. Unexpectedly, most GSLs showed no association between their levels and the enzyme activity that catabolizes them. Genomic mapping identified 30 shared predicted modifier genes between the enzymes and GSLs, which are clustered in three pathways and are associated with other diseases. Surprisingly, they are regulated by ten common transcription factors, and their majority by miRNA-340p. In conclusion, we have identified novel regulators of GSL metabolism, which may serve as therapeutic targets for LSDs and may suggest the involvement of GSL metabolism in other pathologies.

## 1. Introduction

Hydrolytic enzymes are abundant in lysosomes [1]. In a healthy cell, the biosynthesis and catabolism of macromolecules are subject to regulatory mechanisms that maintain cellular homeostasis [2]. The degradative processes in lysosomes are controlled by their own enzymes [3,4]. Lysosomes play a central role in several biological processes, including energy metabolism, signaling, plasma membrane repair, secretion, and others [3]. Loss-of-function variants in genes encoding lysosomal proteins cause lysosomal storage disorders (LSDs), a group of diseases characterized by intracellular buildup of partially degraded material [5]. Growing evidence suggests that variants in lysosomal genes increase the risk of developing Parkinson’s disease (PD) [6,7].

In the sphingolipidoses, a subset of LSDs, glycosphingolipids (GSLs) accumulate in late endocytic organelles (late endosomes/lysosomes) and participate in their pathological cascades [8]. Current treatments for LSDs include substrate reduction therapy (SRT), which aims to reduce the rate of biosynthesis of stored substrates [5,9,10], and enzyme replacement therapies (ERT) aimed at replacing a deficient enzyme [11,12]. Emerging treatments include gene and cell therapies [13,14,15] and chaperones for improving enzyme folding and trafficking [16]. Although there is a range of therapeutic options for LSDs, they have limitations, such as tissue accessibility [17], antibody-mediated reaction [18], cost [19], and others. So far, therapies aimed at increasing enzyme activity or reducing lipid levels by modulating a second (modifier) gene have not been studied. In this context, a deeper understanding of the regulatory mechanisms that govern GSLs metabolism must be uncovered to fully develop this approximation.

Genome-wide association studies (GWAS) in humans and systems genetics strategies, which include gene mapping in model organisms, have identified genetic regulators of physiological and pathophysiological processes [20,21,22]. The Hybrid Mouse Diversity Panel (HMDP) has been a useful tool because genomes and tissue transcriptomes are freely available, allowing the combination of modifier gene mapping by GWAS and pathway analysis [23,24]. In this study, we have analyzed the activities of 11 lysosomal enzymes and several of their natural substrates in 25 strains of the HMDP panel followed by gene mapping and transcript integration. We identified a lack of correlation between most enzyme activities and their mRNA levels. Similarly, most substrates had no association between their levels and the enzyme activity that catabolizes them. Finally, we mapped putative modifier genes of each lysosomal enzyme and GSL by GWAS. We found associations between the mRNA levels of many modifier genes and enzyme activities or GSL levels. We clustered the putative modifiers in pathways and identified common and uncommon genetic regulators between GSLs and lysosomal enzymes, including transcription factors that regulate them. Our discoveries may help develop novel therapeutics for diseases with altered lysosomal enzyme activities and GSLs.

## 2. Results

### 2.1. High Variability in the Hepatic Activity of Lysosomal Enzymes across Mouse Strains

**Figure 1:**
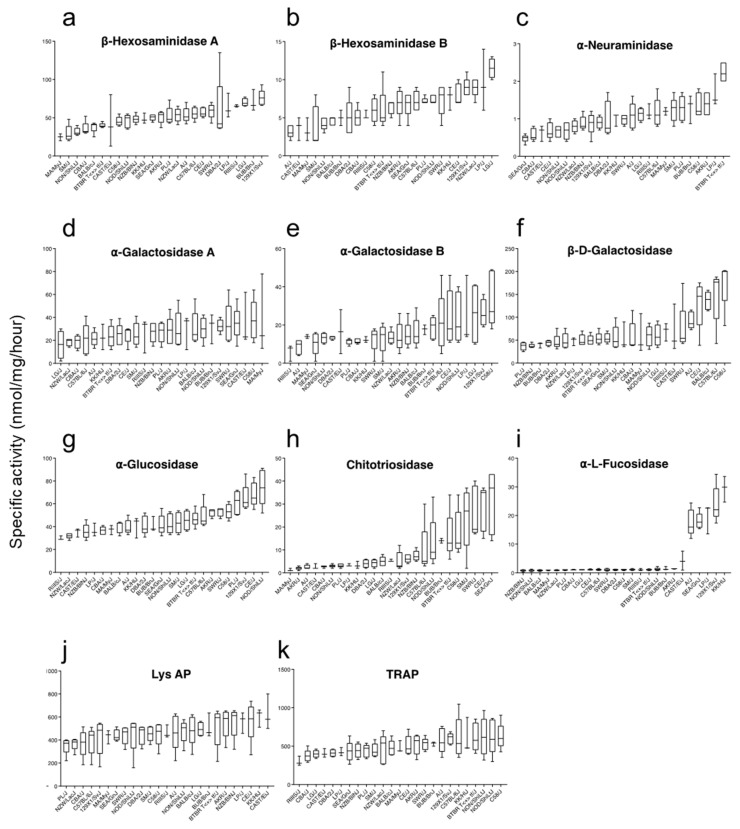
Hepatic variation in lysosomal enzyme activities across inbred strains. Enzyme activity are expressed as nmol/mg/hour. (**a**) Distribution of β-Hexosaminidase A. (**b**) β-Hexosaminidase B. (**c**) α-Neuraminidase. (**d**) α-galactosidase A. (**e**) α-galactosidase B. (**f**) β-D-galactosidase. (**g**) α-Glucosidase. (**h**) Chitotriosidase. (**i**) α-L-Fucosidase. (**j**) Lysosomal acid phosphatase. (**k**) Tartrate-resistant acid phosphatase, activity in the liver of 25 mouse inbred strains. Values are presented as median (n = 3–5 per strain).

We measured hepatic enzyme activity of β-hexosaminidase A and B (defective in Tay-Sachs and Sandhoff disease, respectively), α-neuraminidase (defective in Sialidosis/Mucolipidosis Type I), α-galactosidase A and B (defective in Fabry and Schindler disease), β-D-galactosidase (defective in GM1 Gangliosidosis), α-glucosidase (defective in Pompe), chitotriosidase (elevated in Gaucher disease), α-L-fucosidase (defective in fucosidosis), lysosomal acid phosphatase (elevated in patients with Gaucher), and Tartrate-resistant acid phosphatase (TRAP; altered in Gaucher disease) by fluorimetry in liver samples derived from 25 inbred mice strains using 4-methylumbelliferone (4-MU) based artificial substrates. We observed significant variability in the average enzymatic activity between the different strains (ANOVA *p* ≤ 0.05) (Figure 1). We did not find changes in α-galactosidase A, lysosomal acid phosphatase, and TRAP activities across the tissues analyzed (Figure 1d,j,k). We observed unique activity distribution patterns across the strains for the other enzymes, suggesting specific modifiers for each enzyme.

### 2.2. Lack of Correlation between the Enzyme Activity and Its mRNA Levels

**Figure 2:**
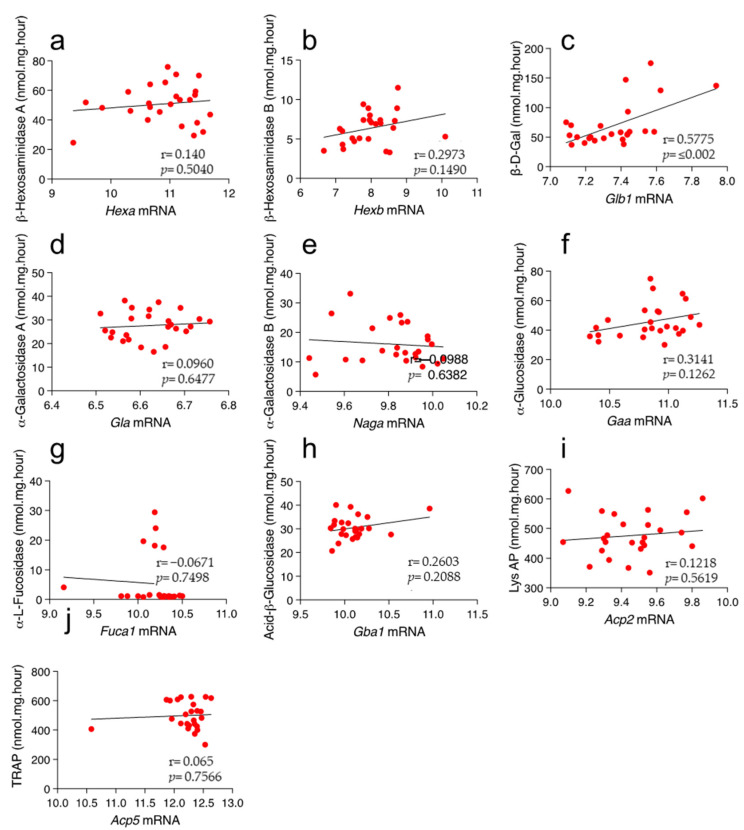
Correlation between expression levels and enzymatic activity in liver of mouse inbred strains. Each dot represents a mouse strain. (**a**) β-Hexosaminidase A. (**b**) β-Hexosaminidase B. (**c**) β-D-galactosidase. (**d**) α-galactosidase A. (**e**) α-galactosidase B. (**f**) α-Glucosidase. (**g**) α-L-Fucosidase. (**h**) Acid-β-glucosidase. (**i**) Lysosomal acid phosphatase. (**j**) Tartrate-resistant acid phosphatase. The Pearson’s correlation was performed using 23 strains of mice. Enzyme activities are expressed as nmol/mg/hour and mRNA levels, which were downloaded from repository GSE16780 UCLA Hybrid MDP Liver Affy HT M430A [24] are expressed as log2 transformed. r, correlation; *p*, *p*-value.

Advantages of using tissues derived from the HMDP panel of inbred mouse strains include the fact that their genomes are sequenced, and transcriptomic data are available. Thus, we analyzed potential correlations between the genes encoding lysosomal enzymes and their activities. Recently we described the natural variation of hepatic acid β-glucocerebrosidase levels across many different mouse strains and included them in this analysis [20]. We did not identify significant correlations between enzyme activity and its transcript levels (Figure 2), with the only exception being *Glb1*, the gene encoding for β-D-galactosidase (r = 0.5775; *p* ≤ 0.002) (Figure 2c). These results indicate that mRNA levels are a poor proxy for enzyme activities.

### 2.3. High Variability in the Hepatic Glycosphingolipid Levels across Mouse Strains

**Figure 3:**
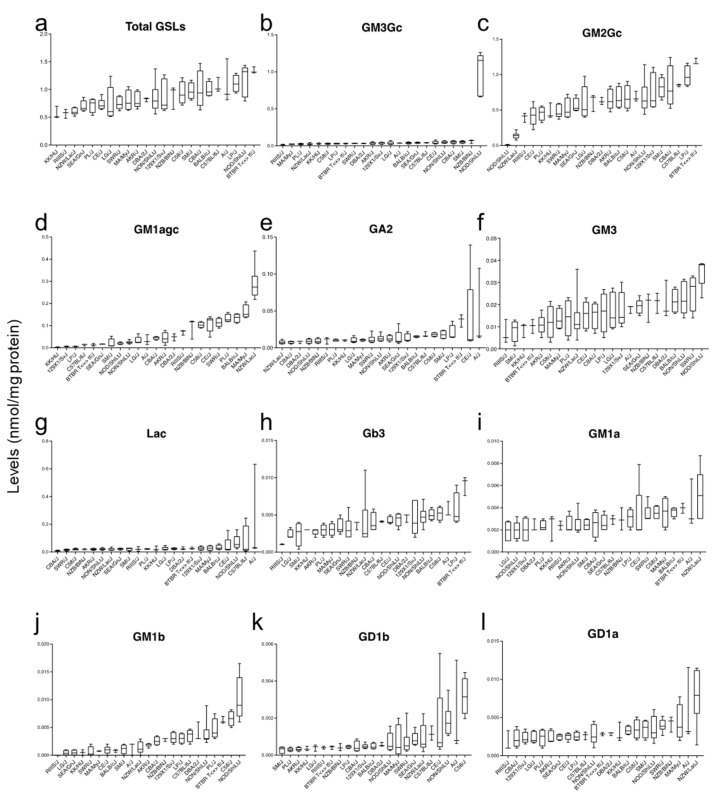
Variation of glycosphingolipids (GSLs) levels in liver of mouse inbred strains. Distribution of glycosphingolipids levels expressed as nmol/mg protein; (**a**) total GSLs (GM3-Gc + GM2-Gc + GM1agc + GA2 + GM3 + LacCer + Gb3 + GM1a + GM1b + GD1b + GD1a) levels. (**b**) GM3-Gc. (**c**) GM2-Gc. (**d**) GM1agc. (**e**) GA2. (**f**) GM3. (**g**) LacCer (Lac). (**h**) Gb3. (**i**) GM1a. (**j**) GM1b. (**k**) GD1b. (**l**) GD1a, in the liver of 23 mouse inbred strains. Values are presented as median (n = 3–5 per strain).

Next, we measured the levels of GSLs in livers of the inbred mice strains in which we had access to enough material for three biological replicates (23/25) by Normal Phase-High-Performance Liquid Chromatography (NP-HPLC). We observed significant variability in GSLs among the strains, especially in total GSLs, GM3-Gc, GM2-Gc, GM1agc, GM3, Gb3, GM1a, GM1b, GD1b, and GD1a (Figure 3). For example, the levels of GM3-Gc were significantly increased (ANOVA *p* < 0.0001) in NOD/ShiLtJ compared with the other samples (Figure 3b). These results indicate that GSLs levels vary across strains.

### 2.4. Correlations between the GSLs and the mRNA Levels of the Biosynthetic Genes

**Figure 4:**
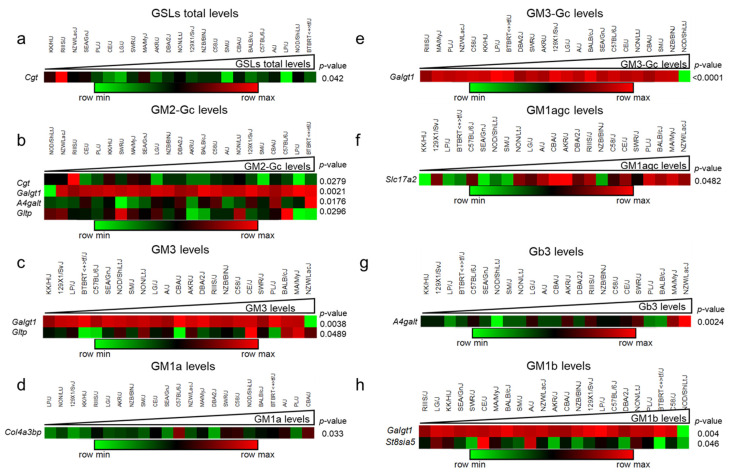
Correlations between GSLs and the mRNA levels of the GSL biosynthetic genes. (**a**) total GSL. (**b**) GM2-Gc. (**c**) GM3. (**d**) GM1a. (**e**) GM3-Gc. (**f**) GM1agc. (**g**) Gb3. (**h**) GM1b levels. Only the genes with significant *p* values with its trait using Pearson’s correlations (*p* ≤ 0.05) were plotted.

**Figure 5:**
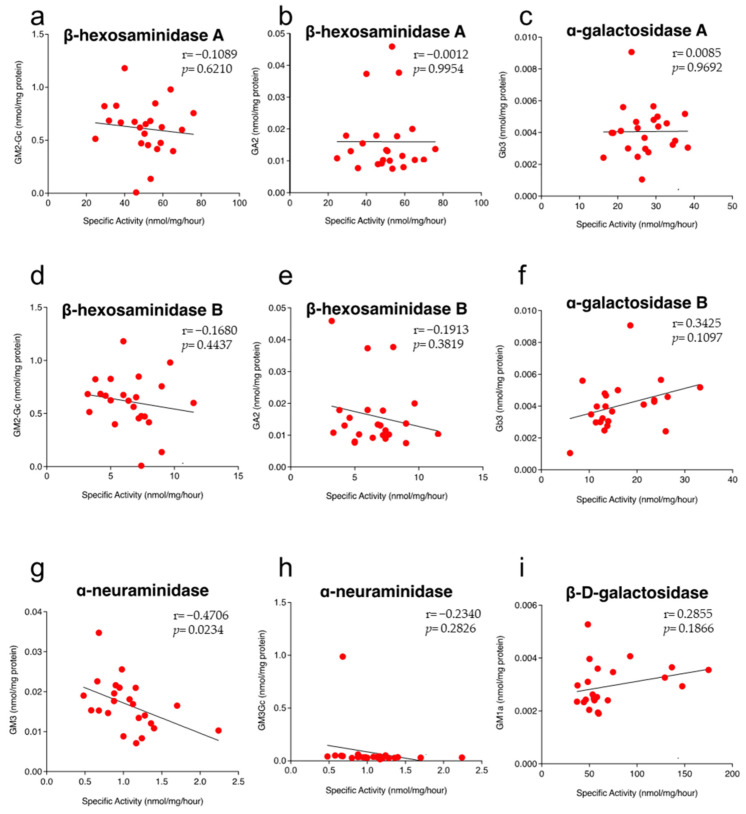
Correlation of hepatic lysosomal enzyme activities and specific substrates levels. Each dot represents a mouse strain. (**a**) HexA vs. GM2-Gc. (**b**) HexA vs. GA2. (**c**) ⍺-Gal A vs. Gb3. (**d**) HexB vs. GM2-Gc. (**e**) HexB vs. GA2. (**f**) ⍺-Gal B vs. Gb3. (**g**) Neu vs. GM3. (**h**) Neu vs. GM3-Gc. (**i**) β-D-Gal vs. GM1a; r, Pearsons’ correlation; *p*, *p*-value.

A possibility is that GSL levels could correlate with their biosynthesis rate. Since we started from frozen tissues, we could not test this directly. Instead, we utilized the transcriptomic data available from the repository GSE16780 UCLA Hybrid MDP Liver Affy HT M430A [24]. We found transcript probes for 21 mRNA of the 21 anabolic enzymes of the GSLs pathway and four GSL transfer proteins. The analyzed gene list of the biosynthetic pathway is presented in the Supplementary Table S1. The expression values were organized according to GSLs levels from lowest to highest and presented as a heatmap. The analysis showed significant correlations for Cgt (r = −0.4263; *p* = 0.042) with total GSLs (Figure 4a). For GM2-Gc with Cgt (r = −0.4582; *p* = 0.0279), Galgt1 (r = 0.6078; *p* = 0.0021), A4galt (r = 0.4903; *p* = 0.0176), Gltp (r = −0.454; *p* = 0.0296) (Figure 4b). GM3 levels correlated with Galgt1 (r = −0.579; *p* = 0.0038), Gltp (r = 0.4151; *p* = 0.0489) (Figure 4c). GM1a with Col4a3bp (r = 0.4458 *p* = 0.033) (Figure 4d). GM3-Gc is associated with Galgt1 (r = −0.9591, *p* ≤ 0.0001) and it was the most significant correlation (Figure 4e). GM1agc levels with Slc17a2 (r = 0.4163; *p* = 0.0482) (Figure 5f). Gb3 with A4galt (r = 0.6011, *p* = 0.0024) (Figure 4g) and GM1b with Galgt1 (r = −0.5764; *p* = 0.004) and St8sia5 (r = −0.4194, *p* = 0.0046) (Figure 4h). No significant correlations were found between the majority of GSLs and biosynthetic genes (Supplementary Table S2); thus, we analyzed potential correlations between GSL levels and the enzyme activity that catabolizes them across the mouse panel.

### 2.5. Lack of Correlation between Hepatic Lysosomal Enzyme Activity and Their Natural Substrates across Mouse Strains

It is possible to speculate that the strains that present high activity of a particular enzyme should have reduced levels of its natural substrate because the enzyme catabolizes it. Unexpectedly, for most enzymes, we did not find significant correlations between the GSL levels and the enzyme activity that degrades it (Figure 5), except for neuraminidase and GM3-Gc (r = −0.4706; *p* = 0.0234) (Figure 5g). These results suggest that for most strains, the rate of biosynthesis and/or uptake of GSLs varies along with the catabolic rates which most likely are genetically regulated.

### 2.6. Identification of Putative Modifier Genes of Lysosomal Enzyme Activity and Sphingolipids Levels

To identify genetic regulators, we conducted genome-wide association studies with a quality control analysis that considered the population structure of the HMDP panel strains to reduce false associations [25,26]. We used enzyme activity levels as a trait and included the β-glucosidase activity, which we reported previously in the same and a few other strains [20]. For all the enzymes together, we identified 211 significant Single Nucleotide Variants (SNVs) that passed the empiric threshold of significance *p* ≤ 4.1 × 10^−6^ (−log10P = 5.39), previously calculated by permutations [21,22,26], while the Bonferroni threshold was *p* ≤ 3.9 × 10^−7^ [26]. These SNVs were located in different genomic regions (exonic, intronic, UTR3, downstream, and intergenic) (Table 1, Supplementary Table S3) in a total of 137 non-redundant genes. Similarly, we identified 3215 SNVs associated with GSLs levels (1744 non-redundant genes) whose variants are located in different genomic regions (Table 1, Supplementary Table S3). These analyses indicated that our strategy has sufficient power to map putative modifier genes.

### 2.7. Correlations between the Traits and the mRNA Levels of Putative Modifiers 

**Figure 6:**
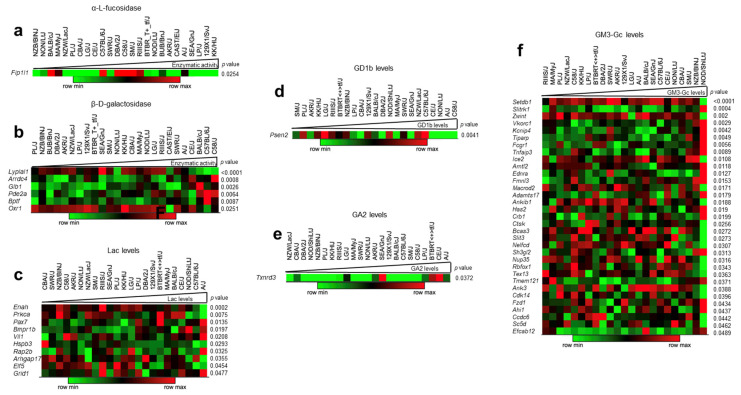
Correlations between the traits (enzyme activities or GSLs) and the mRNA levels of the identified modifier genes. (**a**) α-L-Fucosidase. (**b**) β-D-galactosidase. (**c**) LacCer (Lac). (**d**) GD1b. (**e**) GA2. (**f**) GM3-Gc. Only the genes with significant *p* values with its trait using Pearson’s correlations (*p* ≤ 0.05) were plotted.

To prioritize the putative modifier genes that could regulate each enzyme, we searched for correlations between the transcript levels of putative modifier genes and their traits (enzyme activity and GSL levels, respectively) (Figure 6). We found transcript probes for 67 mRNA of the 137 putative modifiers of the enzymes. The expression values were organized according to enzyme activity from lowest to highest and presented as a heatmap. The analysis showed significant correlations in Fip1l1 (r = −0.4462; *p* = 0.0254) with α-L-fucosidase (Figure 6a). For β-D-galactosidase with Lyplal1 (r = −0.702; *p* = <0.0001), Arrdc4 (r = 0.627; *p* = 0.0008), Pde2a (r = 0.5306; *p* = 0.0064), Glb1 (r = 0.5753; *p* = 0.0026), Bptf (r = 0.5135; *p* = 0.0087), Oxr1 (r = −0.447; *p* = 0.0251) (Figure 6b). No significant correlations were found for the other enzymes analyzed. We used SIFT to explore the impact of genetic variants on the genes identified by GWAS (benign or deleterious changes) associated with changes in enzyme activity [27], because the full genomes of the strains are known [28]. This strategy identified 308 predicted deleterious variants (Supplementary Table S4) in 43 of the 67 genes whose functions are related to organelle biogenesis (Chchd6) [29], intracellular signaling (Pde4dip) [30], and tissue development (Fam181b) [31], among others. These results suggest that amino acid substitution could affect protein function and signaling pathways leading to changes in enzyme activity. 

The same analysis was performed to identify putative modifiers of GSL levels (Figure 6c–f). For 1744 non-redundant SNVs, we found expression values for 994 genes. The analysis identified 45 significant correlations, of which 33 were correlated with GM3-Gc levels, 10 genes with LacCer, and one gene with GD1b and GA2 (Figure 6c–f). Overall, we recorded 4.9% (52/1061) of significant correlations distributed between the two traits. We also explored the impact of genetic variants associated with changes in GSLs with SIFT [27]. This strategy identified 515 deleterious variants predicted to disrupt the protein structure (Supplementary Table S4) in 132 genes related to DNA methyltransferase activity (Setdb1) [32] and synapse (Slitrk1) [33], among others.

### 2.8. Enrichment Analysis and Common Modifier Genes between Glycosphingolipids Levels and Lysosomal Enzyme Activities

**Figure 7:**
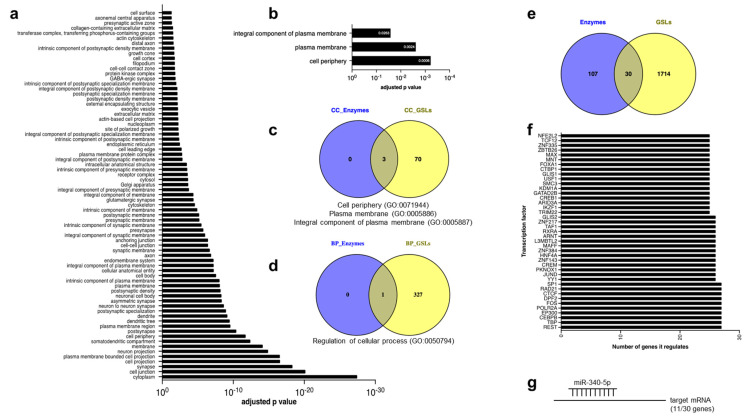
Enrichment analysis and common modifier genes between glycosphingolipids levels and lysosomal enzyme activities. (**a**) Cellular component functional enrichment analysis for gene sets (1744 non−redundant genes) of six substrate (GM3−Gc, GA2, Lac, GM1b, GD1b, GD1a), analyzed by g:Profiler. We found 73 GO_CC associated. (**b**) Cellular component functional enrichment analysis for gene sets (137 non−redundant genes) of eight enzymes (acid β−glucosidase, α−galactosidase A, α−galactosidase B, α−glucosidase, β−D−galactosidase, chitotriosidase, α−L−fucosidase, tartrate−resistant acid phosphatase). (**c**) Venn diagram with common GO terms cellular component between two traits. (**d**) Common GO terms biological processes between two traits. (**e**) Common genetic regulators between two traits. (**f**) Transcription factors that bind to the common genes. (**g**) Cartoon with common genetic regulators that miR−340−5p can bind.

If there is an orchestrated regulation of GSL levels and the enzymes that degrade them, it would be expected to observe enrichment in common pathways [34]. We therefore utilized gProfiler [35] to perform enrichment analysis using the putative modifier genes lists. For the modifier of enzyme activities, we found significantly associated pathways such as cell periphery (*p* = 5.9 × 10^−4^), plasma membrane (*p* = 2.4 × 10^−3^), and integral components of the plasma membrane (*p* = 2.6 × 10^−2^) (Figure 7b), which could be related to endocytic processes necessary to deliver key molecules to the lysosome, including the lysosomal enzymes that can be recycled from the extracellular space. Significant biological processes analysis included regulation of cellular processes (*p* = 3.9 × 10^−2^) (Figure 7d) (Supplementary Table S5). We did not find significant enrichment for the molecular function category. For GSLs, we observed enrichment in terms like cytoplasm (*p* = 3.5 × 10^−28^), cell junction (*p* = 7 × 10^−21^), synapse (*p* = 4.6 × 10^−19^), and 70 other pathways related to cellular components (Figure 7a; Supplementary Table S5). Many of these pathways require cellular membranes, where GSLs play a structural role. Significantly enriched Gene Ontology (GO) terms included protein binding (*p* = 9.1 × 10^−31^), ion binding (*p* = 8.8 × 10^−14^), binding (*p* = 2.3 × 10^−13^), ATP binding (*p* = 9.4 × 10^−13^), carbohydrate derivate binding (*p* = 1.8 × 10^−1^), and 27 other pathways related to molecular functions (Supplementary Table S5). Biological processes terms revealed 328 pathways, including system development (*p* = 4.3 × 10^−39^), anatomical structure development (5.6 × 10^−38^), and multicellular organism development (*p* = 1.4 × 10^−37^). We searched for the overlap between the cellular component domains of modifiers of enzyme activity and GSLs, which resulted in three common pathways (GO:0071944—cell periphery, GO:0005886—plasma membrane, and GO:0005887—integral component of plasma membrane) (Figure 7c) and one pathway associated with biological processes (GO:0050794; regulation of cellular process) (Supplementary Table S5).

### 2.9. Common and Uncommon Modifiers between Hepatic Lysosomal Enzyme Activity and Sphingolipids Levels

Common regulators of GSLs and enzymatic activities are relevant for understanding GSL metabolism and may be attractive therapeutic targets for LSDs. Therefore, we examined the overlap between them. We found 30 common and 1821 uncommon genes (Figure 7e). We explored their functions and identified genes involved in mitochondrial biogenesis and dynamics (Tfb1m, Timen135, Chchd6) [29,36,37], cell proliferation (Fstl5, Fzd10, Arhgap18) [38,39,40], platelet function (Cdh6) [41], vesicular trafficking (Vps45) [42], gene expression (Tfb1m, Zfat) [36,43], and regulating levels of the proto-oncogene MYC (Pvt1) [44]. Many of the 30 genes have been linked to diseases, such as Pvt1, Tiam2, Fstl5, Fzd10, Cdh6, Pvt1, Chchd6 in liver, colorectal, nasopharyngeal, and gastric cancer [45,46,47,48,49,50]. Others participate in neurodegenerative conditions; PD, schizophrenia, and intellectual disability (Tenm4, Pde4dip, Grid2, Arhgap18) [51,52]. These results suggest that lysosomal enzymes and GSLs may play a role in their pathophysiology and should be explored further (Table 2). 

To better understand the molecular regulation of these 30 genes, we analyzed the transcription factors that bind to their promoters and/or enhancers (Figure 7f). We found no information for three of the 30 genes since they are putative (Rik) genes. The following transcription factors can bind to the 27 genes for which we have information: REST, TBP, CEBPB, EP300, POLR2A, FOS, DPF2, CTCF, RAD21, and SP1. Some of these transcription factors are broad regulators of transcription, such as TBP and POLR2A, while others are selective for specific processes, such as CTCF and RAD21. Considering all the promoters/enhancers of the 27 shared genes, we identified a total of 533 transcription factors that can bind them, although some only bind a few genes (Supplementary Table S6). We also searched for potential shared microRNA (miRNA) regulators using miRTarBase, a curated microRNA database [53]. We identified that miR-340-5p can bind to 11 of the 27 known common genes (Tusc1, Fam91a1, Zc3h12c, Adamts5, Tmem135, Tenm4, Grid2, Csnk1g3, Cdh6, Fam181b, and Pde4dip; *p* = 2.2 × 10^−2^) (Figure 7g). This result suggests that miRNA-340-5p regulates GSLs metabolism and may be involved in the pathogenesis of LSDs and the disorders described in Table 2.

**Table 2:** Common genetic modifiers associated with enzyme activity and hepatic glycosphingolipid levels in inbred mouse strains. The references related to this table are presented as supplementary material.

Gene	Description	Traits				Related Functions	Associated Human Diseases	Previosly Associated with Traits	References
		Enzyme	*p*-Value GWAS	GSLs	*p*-Value GWAS				
*Tiam2*	T cell lymphoma invasion and metastasis 2	α-Glucosidase	1.89 × 10^−6^	GM3-Gc	1.51 × 10^−31^	neuroplasticity	liver cancer	No	[47,54]
*Tfb1m*	Dimethyladenosine transferase 1, mitochondrial	α-Glucosidase	1.89 × 10^−7^	GM3-Gc	1.51 × 10^−31^	promotion of mitochondrial biogenesis	deafness	No	[36,55]
*Dok5*	Insulin receptor substrate 6	β-D-galactosidase	1.43 × 10^−7^	Lac	1.38 × 10^−12^	osteoblast differentiation, insulin and IGF-1 signaling	cancer, Alzheimer’s disease	No	[56,57,58,59]
*4930433b08Rik*	RIKEN cDNA 4930433B08 gene	β-D-galactosidase	2.32 × 10^−8^	Lac	1.38 × 10^−12^	-	-	-	-
*A830019l24Rik*	RIKEN cDNA A830019L24 gene	β-D-galactosidase	1.43 × 10^−7^	Lac	1.38 × 10^−12^	-	-	-	-
*Tmem135*	Transmembrane protein 135	β-D-galactosidase	1.43 × 10^−7^	GM3-Gc	1.51 × 10^−31^	involved in mitochondrial dynamics	retinal diseases	No	[37,60]
*Fam181b*	Family with sequence similarity 181, member B	β-D-galactosidase	1.43 × 10^−7^	GM3-Gc	1.51 × 10^−31^	increased expression during mouse development	-	No	[31]
*Tenm4*	Teneurin transmembrane protein 4	β-D-galactosidase	1.43 × 10^−7^	GM3-Gc	1.51 × 10^−31^	cell maturation and myelination in SNC	neuropsychiatric disorders, Parkinson’s disease	No	[51,61,62,63]
*Plk2*	Serine/Threonine-protein kinase PLK2	α-L-Fucosidase	1.43 × 10^−7^	GM3-Gc	1.51 × 10^−31^	cell proliferation, alpha-synuclein phosphorylation	pulmonary fibrosis	No	[64,65]
*Stk32a*	Serine/Threonine kinase 32A	α-L-Fucosidase	1.43 × 10^−7^	GM3-Gc	1.51 × 10^−31^	kinase activity	lung cancer	No	[66,67]
*Dpysl3*	Dihydropyrimidinase like 3	α-L-Fucosidase	1.43 × 10^−7^	GM3-Gc	1.51 × 10^−31^	cell migration, cytoskeletal dynamics and inflammation	gastric cancer, amyotrophic lateral sclerosis	No	[68,69,70]
*Prex1*	PIP3 Dependent rac exchange factor 1	α-L-Fucosidase	6.67 × 10^−8^	GM3-Gc	1.51 × 10^−31^	contributes to the effector activity of mouse neutrophils	prostate cancer	No	[71,72]
*Fstl5*	Follistatin-related protein 5	α-L-Fucosidase	6.67 × 10^−8^	Lac	1.38 × 10^−12^	play a role in cell proliferation	hepatocellular carcinoma	No	[38,73]
*Vps45*	Vacuolar protein sorting-associated protein 45	α-L-Fucosidase	1.13 × 10^−1^	GM3-Gc	1.51 × 10^−31^	vesicle-mediated protein trafficking from the Golgi	neutrophil disorders	No	[42,74]
*Hist2h2be*	Histone cluster 2 H2B family member E	α-L-Fucosidase	1.13 × 10^−1^	GM3-Gc	1.51 × 10^−31^	is necessary for proliferation	breast cancer	No	[75]
*Pde4dip*	Phosphodiesterase 4D interacting protein	α-L-Fucosidase	2.29 × 10^−7^	GM3-Gc	1.51 × 10^−31^	cAMP-dependent pathway to Golgi and/or centrosomes	schizophrenia	No	[52]
*Tusc1*	Tumor suppressor candidate 1	α-L-Fucosidase	6.67 × 10^−8^	GM3-Gc	1.51 × 10^−31^	reduced cell proliferation in vitro *e in vivo*	glioblastoma	No	[76,77]
*Fzd10*	Frizzled class receptor 10	α-L-Fucosidase	6.67 × 10^−8^	GM3-Gc	1.51 × 10^−31^	promotes cell proliferation through Wnt1	cancer	No	[39,48]
*Grid2*	Glutamate ionotropic receptor delta type subunit 2	α-L-Fucosidase	6.67 × 10^−8^	Lac	1.38 × 10^−12^	receptor for glutamate	neurodevelopmental syndrome/intellectual disability	No	[78]
*Zc3h12c*	Zinc finger CCCH-type containing 12C	α-L-Fucosidase	1.64 × 10^−6^	GM3-Gc	1.51 × 10^−31^	RNA stability associated with inflammatory genes	psoriasis	No	[79,80]
*Arhgap18*	Rho GTPase activating protein 18	α-L-Fucosidase	6.67 × 10^−8^	GM3-Gc	1.51 × 10^−31^	role in migration, spreading and controls stress fiber formation	schizophrenia in Chinese population	No	[40,81]
*Cdh6*	Cadherin 6	α-L-Fucosidase	1.34 × 10^−6^	Lac	1.38 × 10^−12^	inhibit platelet aggregation	cancer	No	[41,49]
*Fam91a1*	Family with sequence similarity 91 member A1	α-L-Fucosidase	6.67 × 10^−8^	GM3-Gc	1.51 × 10^−31^	WDR11 complex (vesicular trafficking)	adenocarcinoma	No	[82,83]
*4933412e24Rik*	RIKEN cDNA 4933412E24 gene	α-L-Fucosidase	6.67 × 10^−8^	Lac	1.38 × 10^−12^	-	-	-	-
*A1bg*	Alpha−1B-Glycoprotein	α-L-Fucosidase	6.67 × 10^−8^	GM3-Gc	1.51 × 10^−31^	cell dynamics and acquired immune response	cervical and bladder carcinogenesis	No	[84,85,86]
*Pvt1*	Pvt1 Oncogene	α-L-Fucosidase	6.67 × 10^−8^	GM3-Gc	1.51 × 10^−31^	promotes cell proliferation	cancer	No	[50,87]
*Adamts5*	ADAM Metallopeptidase with thrombospondin type 1 motif 5	α-L-Fucosidase	6.67 × 10^−8^	GM3-Gc	1.51 × 10^−31^	metalloproteinase that remoldels connective tissue	osteoarthritis	No	[88]
*Csnk1g3*	Casein kinase 1 gamma 3	α-L-Fucosidase	3.51 × 10^−7^	Lac	1.38 × 10^−12^	wnt signaling pathway	breast, brain and colon cancer	No	[89,90]
*Chchd6*	Coiled-coil-helix-coiled-coil-helix domain containing 6	Chitotriosidase	1.37 × 10^−6^	GA2	3.27 × 10^−6^	mitochondrial membrane morphology	cancer	No	[29]
*Zfat*	Zinc finger protein ZFAT	TRAP	7.72 × 10^−7^	Lac	1.38 × 10^−12^	immune response	hashimoto’s disease	No	[43,91]

## 3. Discussion

In this study we searched for genetic modulators involved in the regulation of the lysosomal enzyme activities and the levels of substrates related to GSLs, with the idea of finding novel therapeutics targets for disorders in which they participate. By GWASs, we identified common and uncommon genetic regulators, evaluated the associations between modifier gene mRNA levels and each trait, and also clustered them in pathways. We identified 30 shared putative modifiers and described the transcription factors that are predicted to regulate them, and we noted that the miRNA340-5p can bind to 11 of these genes.

Our first unexpected finding was that most lysosomal enzyme activities do not correlate with their mRNA levels, nor with most of their substrate levels. Although enzyme activity can decrease with age [92], we used sex and age-matched samples; thus, the variation observed across strains was shown not to be due to any of these factors.

Another unexpected finding was that GM2-Gc levels correlate with the mRNA levels of the *Cgt* gene, which encodes for the UDP-galactose ceramide galactosyltransferase (CGT). CGT is a key enzyme for the biosynthesis of galactocerebrosides. Gangliosides, including GM2 derivates, are built from glucosylceramide and not from the galacto series [93]. However, for most of the biosynthetic genes there were no associations between the amount of lipids and the transcript levels of their anabolic pathways. Altogether, our results suggest that the GSL biosynthesis rate and uptake differ across the mouse strains, suggesting the existence of specific modifier genes for each trait.

Our third unexpected finding was that TFEB, the master transcriptional regulator of lysosomal genes [94], did not appear in the list of modifiers of lysosomal enzymes. This may be due to the fact that we screened for enzymatic activity instead of mRNA levels, and we showed a lack of correlation between transcript levels and enzyme activity under physiological conditions, at least for most enzymes. One exception was β-D-galactosidase, for which we found a positive correlation between its transcript levels and activity. Furthermore, the GWAS for this enzyme identified *Glb1*, the gene encoding for β-D-galactosidase, as a putative modifier of its activity, validating the power of discovery of our population-based strategy [95].

Our study had some limitations: First, we quantified lysosomal traits from liver homogenates that were not in living or isolated organelles, which may have diluted enzyme activity or promoted molecular interactions that might not occur in vivo because of cellular compartmentalization. Second, we could not directly measure GSLs biosynthesis and uptake because we started with mouse liver samples. Third, we used SNV catalogs with imputation, which may lead to false associations, though with increased mapping resolution. 

Most of the enzymes we assayed are associated with LSDs [5,8]. For many LSDs, no therapies are available, and the few currently available treatments have severe limitations [5]. In this context, targeting a modifier gene could be a novel therapeutic approach. For example, lack of β-D-galactosidase activity triggers GM1 gangliosidosis, a disease with no approved therapies [96]. Our study identified the druggable *Lypla1* and *Pkm* genes as putative modifiers of β-D-galactosidase activity, which can be pharmacologically modulated [97,98]. We found other druggable genes as well for several traits, and with the current gene editing technologies virtually any gene can be targeted. The potential modifying effects of these genes and compounds can be tested in LSDs disease models.

A hallmark of the sphingolipidoses is the intracellular buildup of GSLs, so strategies aimed at reducing their levels could lead us to novel therapies [5,8]. GSLs comprise a ceramide moiety with one or more sugar residues linked to it [99]. An approved therapy for Gaucher and Niemann-Pick disease type C is Miglustat [100,101], a small molecule inhibitor of GSL biosynthesis, thus reducing their levels. Our GSLs GWAS identified more than 50 genes previously associated with sphingolipid metabolism, which served as a positive control, including *B3gnt5, Cln8, Hexb, Pnpla1, St8sia1*, and *Cgt. B3gnt5* regulates GSLs metabolism and lung tumorigenesis [102]. Our study also identified *Lipc* as a modifier of GM3-Gc levels, which has been previously associated with elevated serum levels of liver enzymes (alkaline phosphatase and γ-glutamyl transferase) [103], suggesting a new connection between GM3-Gc and liver damage. Variants in *LIPC, CPS1, PABPC4, CITED2, TRPS1,* and *MVK* are associated with changes in plasma lipoprotein levels [104], connecting novel traits to GSLs metabolism.

Lysosomal leakage has been associated with Alzheimers’ [105], cancer, and inflammation among other conditions [106]. Recently, the phosphoinositide signaling pathway was implicated in lysosomal repair [107]. Many genes of this pathway appear in our discovery list (*Osbpl9, Osbpl6, Pde4dip, Pde2a, Pde1a, Pde7a, Pde7b, Pde4d, Pde8b, Pld5, Pik3r1, Pip4k2a, Pip5k1a, Pip5k1b, Pi4kb, Pdpk1, Atg4c, Atg10*), suggesting that integrity of the lysosomal compartment is key to the proper functioning of enzymes and/or that these enzymes and lipids participate in lysosomal repair. Furthermore, this novel lysosomal repair pathway may facilitate the development of novel therapeutics for these diseases with lysosomal leakage.

Defects in the 30 shared genes are related to several pathologies, such as vision abnormalities (*TMEM135*) [60], cancer (*CDH6* [49], *FZD10* [48], *TIAM2* [47]), neuropsychiatric disorders (*Tenm4* [51], *Pde4dip* [52], *Grid2* [78]), deafness (*TFB1M*) [55], neutrophil disorders (*VPS45*) [74] and others. Lysosomal enzymes and GSLs have been widely studied in cancer and neurodegenerative diseases [46,108,109,110,111]; however, their role in the other identified conditions should be explored.

Although not binding the complete list of shared genes, we identified some transcription factors previously known to be involved in lipid metabolism and autophagy-lysosomal functions (PPARγ, SREBF1, HNF1A, YY1, EGR1, SP1 and TFE3, E2F1, CREB1, MYC) [112,113,114,115,116,117,118,119,120,121], and many more that have not been previously linked to GSL metabolism. We also identified miR-340-5p as a putative regulator of many common modifier genes. Changes in miR-340-5p are linked to preeclampsia, neuroinflammation [122,123,124,125,126], adipocyte differentiation [127], as well as obesity and diabetes [128]. GSL metabolism plays a crucial role in the two last-mentioned disorders, and inhibitors of their biosynthesis have shown promising results in animal models of these conditions, validating the relevance of our strategy [129,130].

In conclusion, we described putative regulators of hepatic lysosomal enzymes and GSLs, many of them druggable and associated with diseases where alterations in GSL metabolism have not been previously described and should be assessed. We expect our findings may facilitate the development of novel therapeutics for conditions with alterations in these traits.

## 4. Materials and Methods

### 4.1. Mouse Tissues

We used 8 weeks-old mice livers derived from 25 inbred mouse strains, which were kindly donated by Dr. Aldons Lusis (University of California, Los Angeles, CA, USA). (i) 129X1/SvJ, (ii) A/J, (iii) AKR/J, (iv) BALB/cJ, (v) BTBR T<+> tf/J, (vi) BUB/BnJ, (vii) C57BL/6J, (viii) C58/J, (ix) CAST/EiJ, (x) CBA/J, (xi) CE/J, (xii) DBA/2J, (xiii) KK/HlJ, (xiv) LG/J, (xv) LP/J, (xvi) MA/MyJ, (xvii) NOD/ShiLtJ, (xviii) NON/ShiLtJ, (xix) NZB/BlNJ, (xx) NZW/LacJ, (xxi) PL/J, (xxii) RIIIS/J, (xxiii) SEA/GnJ, (xxiv) SM/J, (xxv) SWR/J. Tissues were homogenized and adjusted to 50 mg tissue/mL in deionized water with a Potter-Elvehjem tissue homogenizer (Omni International, Kennesaw, GA, USA). Three or more livers per mouse strain were used to quantify traits (Supplementary Table S7).

### 4.2. Enzyme Activity Assays

Lysosomal hydrolase activities were determined using an artificial fluorescent substrate based on 4-methylumbelliferone (4-MU) [131]. For α-glucosidase, 1.47 mM 4-MU α-D-glucopyranoside (Sigma, Dorset, UK) in 100 mM citric acid/100 mM sodium phosphate, 0.1% TritonX-100, pH 4.0 was used as substrate [132]. The substrate for α-galactosidase A and B activities was 5 mM 4-MU α-D-galactopyranoside (Santa Cruz, CA, USA) with and without 250 mM N-acetyl-galactosamine (Sigma, Dorset, UK) in 100 mM citric acid/100 mM tri-sodium citrate, 0.1% TritonX-100, pH 4.0 [133,134]. For measuring β-hexosaminidase A and B activity, 3 mM 4-MU N-acetyl-β-D-glucosaminide (BioChemika, Dorset, UK) in 100 mM citric acid/100 mM sodium phosphate, 0.1% TritonX-100, pH 4.5 was used as substrate. Heat inactivation assay for β-hexosaminidase A was carried out at 50 °C for 3 h [135]. For β-galactosidase activity, 1 mM 4-MU β-D-galactose (Sigma, Dorset, UK) in 200 mM sodium acetate buffer, 100 mM NaCl, 0.1% TritonX-100, pH 4.3 was used as substrate [136]. The substrate for neuraminidase activity was 0.4 mM 4-MU α-D-N-acetylneuraminic acid (Sigma, Dorset, UK) in 0.1 M acetate buffer, 0.1% TritonX-100, pH 4.6 [137,138]. For chitotriosidase activity, 0.013 mM 4-MU chitotrioside (Sigma, Dorset, UK) in 100 mM citric acid/200 mM sodium phosphate, 0.1% TritonX-100, pH 5.2 was used as substrate [139,140]. For total acid phosphatase activity, 5 mM 4-MU phosphate (Sigma, Dorset, UK) with 40 mM NaCl in 200 mM citric acid/200 mM sodium phosphate, 0.1% TritonX-100, pH 4.5 was used as substrate. For tartrate-resistant acid phosphatase (TRAP) activity, 5 mM 4-MU phosphate (Sigma, Dorset, UK) with 40 mM Na Tartrate in 200 mM citric acid/200 mM sodium phosphate, 0.1% TritonX-100, pH 4.5 was used as substrate. The difference between total acid phosphatase activity and TRAP corresponded to lysosomal acid phosphatase (Lys AP) activity [141,142]. The substrate for α-L-fucosidase activity was 60 nM 4-MU α-L-fucopyranoside (Sigma, Dorset, UK) in 200 mM citric acid/200 mM sodium citrate, 0.1% TritonX-100, pH 5.0 [143,144]. We determined the acid-β-glucosidase activity in the same tissues in a previous publication [20], and further analyses were performed here based on the published activity. Liver homogenates were diluted with the buffer corresponding to each enzymatic determination. Three cycles of freezing (liquid nitrogen) and thawing were performed on the samples. Three biological replicates of the diluted liver extracts were incubated with the corresponding substrate at 37 °C for 30 min (or 1 h for α-neuraminidase, β-D-galactosidase, and chitotriosidase). Cold 0.5 M Na_2_CO_3_ (pH 10.7) was added to stop the reaction. Fluorescence intensity in samples was measured in a Synergy HT plate reader (BioTek, Winooski, VT, USA) at 360/460 nm. Protein concentration was measured using a BCA protein assay kit (Thermo Fisher Scientific, New Jersey, NJ USA). Fluorescence values were normalized to protein concentration. A 4-MU standard curve was constructed to calculate specific activity, and the final value was adjusted to one hour of enzymatic reaction.

### 4.3. Glycosphingolipids Levels Quantification

The GSLs were extracted and measured by Normal Phase-High-Performance Liquid Chromatography (NP-HPLC) following published methods [145]. Briefly, the aqueous tissue extract was homogenized in chloroform/methanol (C:M) (1:2 *v*/*v*) and kept overnight at 4 °C. Then, the extracts mixture was centrifuged at 3000 rpm for 10 min at room temperature. We added 0.5 mL of PBS and 0.5 mL of chloroform to the supernatant followed by a 3000-rpm centrifugation for 10 min at room temperature. The lower phase was carefully removed and dried under a stream of nitrogen gas (N2) in a heating block (42 °C), resuspended in 40 μL C:M 1:3 *v*/*v* and mixed with the upper phase. Afterwards, glycosphingolipids-derived oligosaccharides were purified from the samples using C18 columns (Telos, Kinesis, UK) previously pre-equilibrated with 1.25 mL methanol (four times) and 1.25 mL deionized water (three times). We loaded the mixed phase (lower/upper) onto a column and rinsed the sample tube with 1 × 1 mL of deionized water. Then, the C18 column was washed with 4 × 1.25 mL deionized water and eluted it with 1 × 1 mL (C:M) (98:2 *v*/*v*), 2 × 1 mL (C:M) (1:3 *v*/*v*), 1 × 1 mL methanol. The eluates were dried under N2 current and digested with a recombinant Endoglycoceramidase I (rEGCaseI) (GenScript, Oxford, UK) in buffer 50 mM sodium acetate, pH 5.0, 0.6% TritonX-100 (4 μL enzyme + 86 μL buffer) at 37 °C for 16 h. The released glycans were labeled with 310 μL of labelling mix (30 mg/mL anthranilic acid (2AA) and 45 mg/mL sodium cyanoborohydride) in 4% sodium acetate, 2% boric acid in methanol, and heated at 80 °C. Then, we cooled the samples and mixed them with 3 × 1 mL acetonitrile: deionized water (97:3) (*v*/*v*) and added them to a Discovery DPA-6S-SPE tube (Supelco, PA, USA), pre-equilibrated with 1 × 1 mL acetonitrile, 2 × 1 mL deionized water, and 3 × 1 mL acetonitrile. The columns were cleaned with 3 × 1 mL acetonitrile: deionized water (95:5) (*v*/*v*), and the tubes were washed with 2 × 1 mL acetonitrile: deionized water (95:5) (*v*/*v*) and eluted in 0.6 mL deionized water. We took 60 μL from 0.6 mL sample eluted, added 140 μL acetonitrile, and injected 50 μL of this mix (deionized water: acetonitrile) (30:70) (*v*/*v*) onto NP-HPLC (Waters Alliance 2695 separations module and multi-fluorescent detector set at Ex 360/Em 425 nm). To calculate molar quantities from peaks in the chromatogram, we included a calibration standard containing 2.5 pmol 2AA-labelled chitotriose (Ludger, Oxford, UK) for each NP-HPLC run [145]. The chromatographic data were processed using Waters Empower software 3 (Waters, Milford, MA, USA). Fluorescence values by sample were normalized to protein content using a BCA Assay kit (Merck KGaA, Darmstadt, Germany).

### 4.4. Genome-Wide Association Studies (GWAS)

We used the genotype of each strain, and the enzymatic activity or substrate as trait, and its kinship matrix to perform the GWAS using The Efficient Mixed Model Association (EMMA) v.1.1.230 in the R package [26,146]. We used PLINK to remove SNVs in linkage disequilibrium to avoid false associations [25], considering an R^2^ = 0.25, leaving 127,285 independent variants out of the initial four million variants downloaded from the mouse HapMap reference panel (http://mouse.cs.ucla.edu/mousehapmap/full.html, accessed on 28 September 2020) [147].

### 4.5. Gene Expression Array and Heat Maps

For gene expression correlations, we obtained inbred mouse hepatic transcript data from the repository GSE16780 UCLA Hybrid MDP Liver Affy HTM430A [24]. The mRNA levels in the repository were expressed as log2 transformed and were calculated from the Affimetrix chip with the robust multiarray average (RMA) method. To plot the heatmaps, we used Morpheus software (https://software.broadinstitute.org/morpheus, accessed on 15 February 2022).

### 4.6. Functional Impact of Genomic Variants

The functional impact of genomic variants was assessed using the Sorting Intolerant From Tolerant (SIFT) software (https://sift.bii.a-star.edu.sg/www/SIFT_dbSNP.html, accessed on 12 July 2022) [27].

### 4.7. Enrichment Analysis

We used gProfiler [35] with the default settings to perform the pathway enrichment analyses.

### 4.8. Identification of Transcription Factors

We consulted the GeneHancer (GH) database, a catalogue of genome-wide enhancer-to-gene and promoter-to-gene associations, through GeneCards^®^ (https://www.genecards.org/Guide/GeneCard, accessed on 6 September 2022) [148]. Only transcription factors with a significative GH Score were considered.

### 4.9. Statistics

We used Student’s *t*-test, ANOVA with Bonferroni correction, and Pearson correlation. All tests were two-tailed. The significance was considered to be *p* < 0.05. We used an R package [146] and Prism v9.1.0 (GraphPad software, San Diego, CA, USA) for these analyses.

## Figures and Tables

**Table 1:** Summary of the putative modifier genes of enzymatic activity and GSLs levels identified by GWAS.

	Trait	Gene	Region	Chr	Position	Ref	Alt	*p*-Value	SNV *p* < 10^−6^	Non-Redundant Genes
lysosomal enzymes	α-Galactosidase A	*Stard4*	intergenic	18	33494519	C	T	2.88 × 10^−6^	1	1
	α-Galactosidase B	*Barhl2*	intergenic	5	106880801	T	C	4.10 × 10^−7^	1	1
	GCase	*Dmrtc2*	UTR3	7	25662483	A	G	7.46 × 10^−7^	2	2
		*Arhgef1*	UTR3	7	25711350	G	T	7.46 × 10^−7^		
	α-Glucosidase	*Tiam2*	intergenic	17	3338741	T	C	1.89 × 10^−6^	3	2
		*Tfb1m*	intronic	17	3557483	G	T	1.89 × 10^−6^		
	β-D-Galactosidase	*Lyplal1*	intergenic	1	188026657	A	G	9.09 × 10^−9^	88	70
		*4930433B08Rik*	intergenic	3	18512557	A	G	2.32 × 10^−8^		
		*Peak1*	intronic	9	56165236	T	C	2.32 × 10^−8^		
		*Imp3*	intergenic	9	56793621	A	G	2.32 × 10^−8^		
		*Scamp2*	intronic	9	57409841	T	C	2.32 × 10^−8^		
		*Loxl1*	intergenic	9	58188292	A	G	2.32 × 10^−8^		
		*1700072B07Rik*	intergenic	9	58256079	G	A	2.32 × 10^−8^		
		*Arih1*	intergenic	9	59348484	C	T	2.32 × 10^−8^		
		*Pkm*	intronic	9	59506197	A	T	2.32 × 10^−8^		
		*Iqch*	intronic	9	63413504	A	G	2.32 × 10^−8^		
	Chitotriosidase 1	*Wdr89*	intergenic	12	76773815	T	C	2.45 × 10^−8^	8	3
		*Syne2*	intronic	12	76961077	T	C	2.45 × 10^−8^		
		*Chchd6*	intergenic	6	89566833	A	G	1.37 × 10^−6^		
	α-L-Fucosidase	*Myom3*	intergenic	4	135400588	C	T	6.06 × 10^−17^	103	56
		*Vps45*	intronic	3	95807768	C	T	1.13 × 10^−1^		
		*Hist2h2be*	downstream	3	96027761	G	A	1.13 × 10^−1^		
		*Tet2*	intergenic	3	133254547	A	C	1.13 × 10^−1^		
		*Zfp46*	UTR3	4	135847850	A	G	1.13 × 10^−1^		
		*Hnrnpr*	intergenic	4	135915162	C	T	1.13 × 10^−1^		
		*E2f2*	UTR3	4	135750026	C	T	8.13 × 10^−9^		
		*Stkld1*	intronic	2	26790736	A	C	6.67 × 10^−8^		
		*Xkr7*	intergenic	2	152887679	G	A	6.67 × 10^−8^		
		*Ttpal*	intronic	2	163432431	A	G	6.67 × 10^−8^		
	TRAP	*Zfat*	intronic	15	68115989	A	C	7.70 × 10^−7^	5	2
		*Mir30d*	intergenic	15	68244382	C	T	7.72 × 10^−7^		
GSLs	GD1a	*Ctnnbl1*	intronic	2	157632357	T	A	1.46 × 10^−7^	37	26
		*Rap2b*	intergenic	3	61765728	A	C	1.46 × 10^−7^		
		*Arhgef26*	intronic	3	62232093	C	T	1.46 × 10^−7^		
	GA2	*Dars*	intergenic	1	130350640	C	T	1.09 × 10^−11^	190	103
		*Abca16*	intronic	7	127596308	G	A	1.09 × 10^−11^		
		*E130201H02Rik*	intergenic	7	127763574	G	A	1.09 × 10^−11^		
		*Vwa3a*	intronic	7	127887001	G	A	1.09 × 10^−11^		
		*Eef2k*	intronic	7	127993389	A	G	1.09 × 10^−11^		
	LacCer	*Tgs1*	intergenic	4	3571870	C	G	1.38 × 10^−12^	1152	723
		*Dtnb*	intronic	12	3586440	C	T	1.38 × 10^−12^		
		*Lyn*	intronic	4	3673421	A	G	1.38 × 10^−12^		
		*Ghr*	intergenic	15	3696333	T	C	1.38 × 10^−12^		
		*1810055G02Rik*	intergenic	19	3731017	G	A	1.38 × 10^−12^		
		*Bambi*	intergenic	18	3826103	C	A	1.38 × 10^−12^		
		*Hnf4g*	intergenic	3	3989664	G	T	1.38 × 10^−12^		
		*Dnajc27*	UTR3	12	4106955	G	C	1.38 × 10^−12^		
		*Mterf1b*	intergenic	5	4503200	A	G	1.38 × 10^−12^		
		*Impad1*	intergenic	4	4885958	C	G	1.38 × 10^−12^		
	GD1b	*Ahctf1*	intergenic	1	181812047	C	A	1.03 × 10^−8^	23	17
		*Psen2*	intronic	1	182170093	C	T	1.03 × 10^−8^		
		*Fhit*	intronic	14	11843484	G	A	1.03 × 10^−8^		
	GM3Gc	*Cdk6*	intergenic	5	3011917	T	C	1.51 × 10^−31^	1811	995
		*Insr*	intergenic	8	3058687	C	T	1.51 × 10^−31^		
		*Eif4enif1*	intronic	11	3143753	G	A	1.51 × 10^−31^		
		*Tiam2*	intronic	17	3417745	T	C	1.51 × 10^−31^		
		*Ppp6r3*	intronic	19	3539614	C	T	1.51 × 10^−31^		
		*Tfb1m*	intronic	17	3540913	C	A	1.51 × 10^−31^		
		*1700102H20Rik*	intergenic	17	3611518	G	A	1.51 × 10^−31^		
		*Pex1*	intronic	5	3632859	T	G	1.51 × 10^−31^		
		*Ankib1*	intronic	5	3711311	C	T	1.51 × 10^−31^		
		*Ankib1*	intronic	5	3778272	C	T	1.51 × 10^−31^		
	GM1b	*Hrasls*	intergenic	16	29161604	A	C	2.71 × 10^−6^	2	1

## Data Availability

We obtained inbred mouse hepatic transcript data from the repository GSE16780 UCLA Hybrid MDP Liver Affy HTM430A reported in [24].

## Supplementary Materials

The following supporting information can be downloaded at: https://www.mdpi.com/article/10.3390/ijms24054915/s1.
